# Optical Temperature Control Unit and Convolutional Neural Network for Colorimetric Detection of Loop-Mediated Isothermal Amplification on a Lab-On-A-Disc Platform

**DOI:** 10.3390/s19143207

**Published:** 2019-07-20

**Authors:** Da Ye Seul Lim, Moo-Jung Seo, Jae Chern Yoo

**Affiliations:** College of Information and Communication Engineering, Sungkyunkwan University, Suwon, Gyeonggi-Do 440-746, Korea

**Keywords:** lab-on-a-disc, loop-mediated isothermal amplification, point-of-care testing, hydroxy naphthol blue, convolutional neural network

## Abstract

Lab-on-a-disc (LOD) has emerged as a promising candidate for a point-of-care testing (POCT) device because it can effectively integrate complex fluid manipulation steps using multiple layers of polymeric substrates. However, it is still highly challenging to design and fabricate temperature measurement and heating system in non-contact with the surface of LOD, which is a prerequisite to successful realization of DNA amplification especially with a rotatable disc. This study presents a Lab-on-a-disc (LOD)-based automatic loop-mediated isothermal amplification (LAMP) system, where a thermochromic coating (<~420 µm) was used to distantly measure the chamber’s temperature and a micro graphite film was integrated into the chamber to remotely absorb laser beam with super high efficiency. We used a deep learning network to more consistently analyze the product of LAMP than we could with the naked eye. Consequently, both temperature heating and measurement were carried out without a physical contact with the surface of LOD. The experimental results show that the proposed approach, which no previous work has attempted, was highly effective in realizing LAMP in LOD.

## 1. Introduction

Many bacteria are the main causes of food poisoning even though different strains for the same bacteria have different levels of pathogenicity [1]. According to US Centers for Disease Control and Prevention (CDC) 2018 reports, the causing portion of foodborne illness by virus (46%) and bacteria (47%) were similar. However, the rate of hospitalization and death rates of foodborne illnesses by bacteria (88%, 92% for each) were much greater than that by virus (9%, 5%) [2]. The US Food and Drug Administration (FDA) reported that salmonellosis (61%) was the most common outbreak of foodborne illness in the last 3 years (2017–2019) followed by *E-coli O157:H7* infection (17%) [3].

Many researchers have attempted to develop simple methods to screen these food poisoning pathogens. Various types of micro total analysis systems (μTAS) have been developed for miniaturized analysis of diagnostic samples [4]. These disposable integrated biochips of various materials, which have benefits that enable compact-sized fabrication, automation, and low manpower consumption [5,6] are called ‘lab-on-a-chip (LOC)’. Although LOC is a useful biochemical fluidic analysis platform, it has drawbacks such as the need for microscale channels [7], which require complicated fabrication steps that make them difficult to produce at low cost or the need for an external power source connected with wires, which makes the apparatus bulky. Zeng et al. have introduced the high-performance microarray disc, but its use is limited to a stable position because it requires many wired connections [8]. Liu B et al. have suggested a simplified Polydimethylsiloxane (PDMS) pump–chip system with compact size. However, this method is only driven by a single pressure source, which limits fluid control by varying the pressure level [9]. One type of LOC, lab-on-a-disc (LOD), is preferred because it is easy to integrate many fluidic functions using centrifugal, inertial, and Coriolis forces in a single disc, and is operated using only one rotor [10]. When valves and simple external devices are added to the LOD, a wide variety of fluid control operations, such as mixing and positioning, can be implemented within a disc automatically [11,12]. There have been many studies performed using LOD for DNA extraction, protein detection [13], fluorescence immunoassays [14], nutrient determination in water [15], ELISA [16], and foodborne pathogen detection [17]. Each LOD is fabricated to fit to the purpose of the study; however, LODs are not constructed to automatically control the temperature using non-contact sensors. A similar attempt was made by many researchers using LOD. Udin et al. [18] suggested a portable endpoint loop-mediated isothermal amplification (LAMP) detection system, but the heating module is not equipped inside its device and it was limited to detecting LAMP product in a device. Ahmed Sayad et al. [17] proposed lab-on-a-disc LAMP detection system with non-contact condition, but the system used a hot-air gun as the heating source, which leads low energy efficiency.

There are numerous nucleic acid amplification methods, starting with the polymerase chain reaction (PCR) [19,20]. Although PCR is widely used in nucleic analysis, its drawback is that it has to be performed at three temperatures—denaturation (94–95 °C), annealing (55–72 °C), and elongation (72–75 °C) [21]—requiring precise temperature control equipment, which is difficult to fabricate in a compact point-of-care testing (POCT) module and requires a lot of energy for heating and cooling. As an alternative to temperature conversion amplification methods, many isothermal amplification methods have been published in the literature. These include helicase-dependent isothermal DNA amplification (HDA) [22], isothermal multiple displacement amplification (iMDA) [23], isothermal strand displacement amplification (iSDA) [24], nucleic acid sequence-based amplification (NASBA) [25], rolling circle amplification (RCA) [26], recombinase polymerase amplification (RPA) [27], single primer isothermal amplification (SPIA) [28], and loop-mediated isothermal amplification (LAMP) [29].

Among all the isothermal amplifying methods reported to date, the LAMP method has the great benefit that it provides many types of detection methods [30], including a fluorescent detection method using DNA intercalating dye [18] in any nucleic acid amplification method and naked eye visualization using an indicator, which changes color by bonding or dissociating with the by-products produced in the LAMP reaction. Naked eye detection is possible in LAMP because the amount of amplified product and by-product is much larger than PCR [31]. Hydroxy naphthol blue (HNB), a metal-ion indicator, can easily visualize the product of the LAMP reaction by changing color from indigo to sky-blue upon losing a divalent magnesium cation (Mg^2+^) to form the Mg_2_P_2_O_7_ complex [32]. HNB colorimetric naked-eye detection has the great benefit of low cost because it does not need a transilluminator and the price of the reagent is 260 times cheaper than SYBR Green I and 10 times cheaper than EvaGreen [33].

In this study, we propose a temperature measurement and heating system that works in a completely non-contact way: we have used a thermochromic ink to distantly measure the chamber temperature and then integrated a micro graphite film into the chamber to locally absorb laser beams with super high efficiency, using its great thermal conductivity to heat the chamber without contact. Temperature sensing and the colorimetric detection of the LAMP product was carried out using a convolutional neural network.

We validated our work by showing the gel electrophoresis analysis and HNB colorimetric results of the amplified product and compared the data with a conventional method using a general heating incubator. We expect our new system will enable a significant advancement in total automatic nucleic acid analysis.

## 2. Materials and Methods

### 2.1. Disc Fabrication

**Figure 1:**
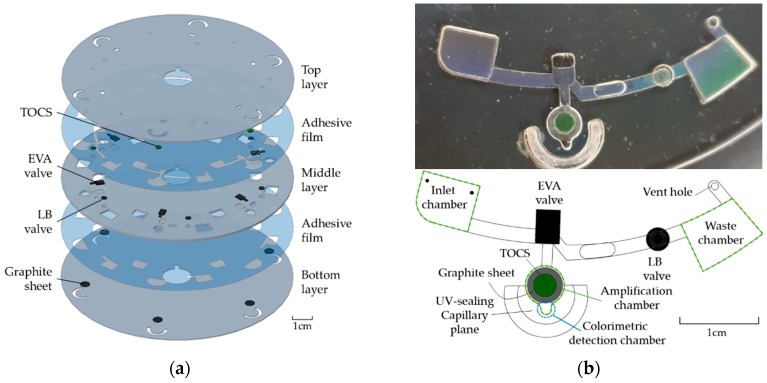
Disc figure; (**a**) disassembled disc, (**b**) top view.

The disc used for the LAMP reaction was prepared using a compact disc-sized polycarbonate (PC) sheet, which was 120 mm in diameter. The whole disc was made using three layers of PC sheets and two layers of adhesive film (Tesa 4928, TESA^®^, Hamburg, Germany). The top and bottom layers were made with 0.6 mm of thickness, for the roles of the lid and bottom. The middle layer was 1.2 mm of thickness, which consisted of the functional chambers and valves (Figure 1a).

The PC layer was shaped using a computer numerical control machine (PromillEZ200, PROTEK Co., Daejeon, Korea) and the adhesive film layer was combined using a 2-sided adhesive film processed by a laser cutting machine (LS100, Gravograph, Rillieux-la-Pape, France).

We attached a 3pi thermo-chromatic sheet (TOCS) at the top of the amplifying chamber. The TOCS made direct contact with the inside of the amplifying chamber. Therefore, it was possible to directly analyze the sample temperature. Furthermore, it was a non-prohibiting material during sample amplification.

We used a laser bursting (LB) valve as the opening valve, which was originally blocking the pathway, but opened when the laser beam was irradiated, to hold the sample reagent before metering. The channel starts at the top adhesive film and leads to the bottom adhesive film with the valve as the fiducial point. The blocking valve was constructed of ethylene-vinyl acetate copolymer (EVA), which melts at ~70 °C.

The EVA valve is easily melted by light-induced heating because of its black color. We placed the EVA valve as the closing valve to seal the amplification chamber and prevent evaporation during heating. When the EVA valve was melted by the laser beam, the disc was immediately centrifuged at 10,000 rpm to block the channel in a radial manner. The UV-sealing capillary plane was prepared so that the wall of the chamber did not burst due to high pressure during centrifugation at 10,000 rpm. UV curing was performed by injecting a UV resin through the capillary plane. The liquid-phase EVA valve has the benefit of completely blocking the entrance of the amplification chamber (Figure 1b). The EVA valve is necessary to prevent the liquid from evaporating and disappearing during the amplification process. Both the LB and EVA valve were validated in previous studies reported by our group [34,35].

We fabricated a micro graphite film (thickness of 50 µm) at the bottom of the amplification chamber, which was used as a heating source by absorbing the laser beam underneath the disc. The micro graphite film has great heat conductivity (400–500 W/mk), which is better than copper and aluminum. However, unlike copper or aluminum, the micro graphite film is darkly colored to effectively absorb laser light and transmit heat to the chamber.

### 2.2. Experimental Apparatus and Driving Protocol

**Figure 2:**
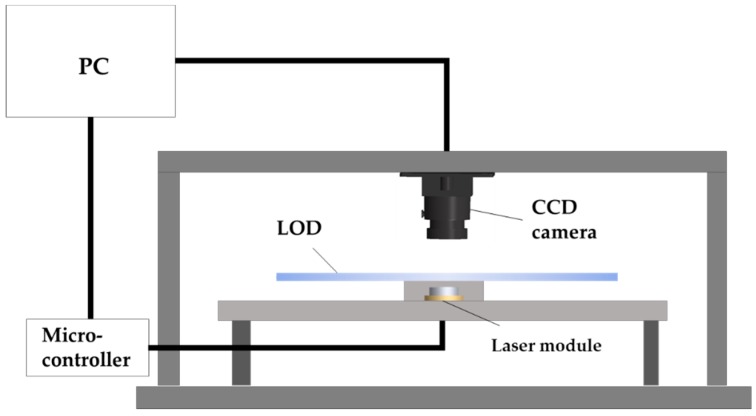
Schematic representation of the data acquisition and analysis module.

The experimental module (Figure 2) was constructed to carry out automatic sample metering, sealing, heating, and temperature control in the LAMP reaction. The base frame was constructed using part of a disassembled conventional disc drive (1328F208370L DVD-W-LF, Toshiba Samsung Storage Technology Co., Suwon, Korea) since the experimental disc is compatible with a standard compact disc drive. One of the main parts was the laser module (QL80T4HD-Y, QSI, Cheonan, Korea, Typical wavelength: 808 nm), which was fabricated to irradiate an infrared beam and used as the driving source of the valves and heating source for the amplification chamber. The other part contained a CCD camera connected to a PC, which was used to detect the temperature via TOCS attached to the top of the amplification chamber and to analyze the endpoint color of the LAMP reagent in colorimetric detection chamber.

**Figure 3:**
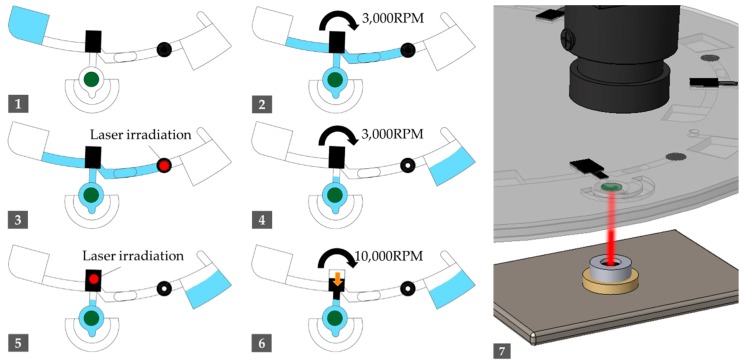
Disc driving sequence indicating the current flow of the fluid (sky-blue). The number index indicates each step 1-7 of Table 1.

The disc was operated using the process reported in Table 1 and Figure 3. To meter an approximate amount of reaction buffer in step 3 and 4, we irradiated the infrared laser for 10 s (at 1.02 W) and rotated the LOD at 3000 rpm to flow out any residual reagent. We irradiated the laser using pulse-width modulation (PWM) to heat the amplification chamber for 40 min to maintain the target temperature (60 °C) during the LAMP reaction.

### 2.3. Sample Preparation and DNA Extraction

We chose *Salmonella Typhimurium* as our target pathogen, the most frequent cause of foodborne illness globally [36]. We cultured one colony of *Salmonella Typhimurium* (IB 5379 Salmonella Typhi. (Tanzania), original ID 73-37671) in tryptone soya agar medium (CM0131, OXOID, Basingstoke, UK). A single colony of the strain was added to tryptone soya broth (CM0876, OXOID, Basingstoke, UK) and incubated overnight at 37 °C to reach a final concentration of 10^8^ CFU/mL (OD_600_ 1.0). Briefly, 1 mL aliquots were prepared for each sample and harvested by centrifugation at 13,000 rpm for 1 min. Subsequently, 900 μL of the supernatant was removed to obtain the cell pellet. The cell pellet was re-suspended in 900 μL of sterilized PBS and the harvesting step was repeated to obtain a 100 μL cell pellet, which contained almost all of the bacterial cells in one aliquot. A sample DNA template was prepared using an Accuprep DNA extraction kit (K-3032, Bioneer, Daejeon, Korea) according to the manufacturer’s protocol using the enclosed reagents, including isopropanol. We used the samples, which are serially diluted 10-fold, 3 times (with the initial 10^8^ CFU/mL) to cover various concentrations of samples present in the real-world [37].

### 2.4. LAMP Reaction

The LAMP reaction mixture was set based on following reagents: 10X Isothermal Amplification Buffer II (B0374S, New England Biolabs, USA, 1X Buffer components: 20 mM Tris-HCl, 10 mM (NH_4_)_2_SO_4_, 150 mM KCl, 2 mM MgSO_4_, and 0.1% Tween^®^ 20. pH 8.8 at 25 °C), 6 mM MgSO_4_ (8 mM total), dNTP Mix for 1.4 mM each (11 969 064 001, Roche, Basel, Switzerland), 1.6 μM of FIP/BIP primers, 0.2 µM of F3/B3 primers, 0.8 μM of LoopF/LoopB primers, 8U of Bst 3.0 DNA Polymerase (M0374S, New England Biolabs, Ipswich, USA), and 120 µM of HNB (H007-10, Dojindo, Kamimashiki, Japan) in a total volume of 25 µL following the manual instructions. In reference to Cho et al. [38], the primer set was prepared using the following sequences, targeting the *invA* gene: 

5′-ATGATGCCGGCAATGGCGTCAGCCAGCTTTACGGTTCCT-3′ (FIP),

5′-GATGACCCGCCATGGTATGGATCCATCACCGATGGTCAGC-3′(BIP),

5′-TTGATAAACTTCATCGCACCGT-3′(LF),

5′-TTATCCTCCGCGCTGTCTACTTA-3′ (LB),

5′-GAAGCGTACTGGAAAGGGAA-3′ (F3), 

5′-GGGATCTGGGCGACAAGA-3′ (B3). 

The LAMP reaction was performed in the amplification chamber at 60 °C for 40 min. The LAMP product was visualized using HNB, which changes color from indigo to sky-blue upon losing Mg^2+^ ion to form the LAMP by-product, Mg_2_P_2_O_7_.

### 2.5. Executing Program for Temperature Control and Colorimetric Analysis of the LAMP Reaction

**Figure 4:**
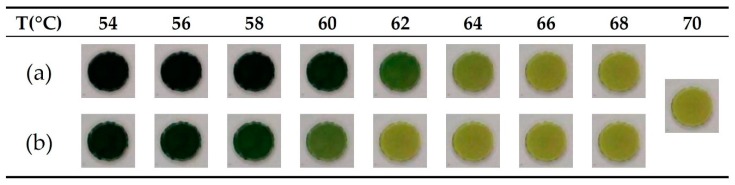
Intensity variation of 3pi thermo-chromatic sheet (TOCS) with temperature during (**a**) initial heating step and (**b**) the cooling step.

**Figure 5:**
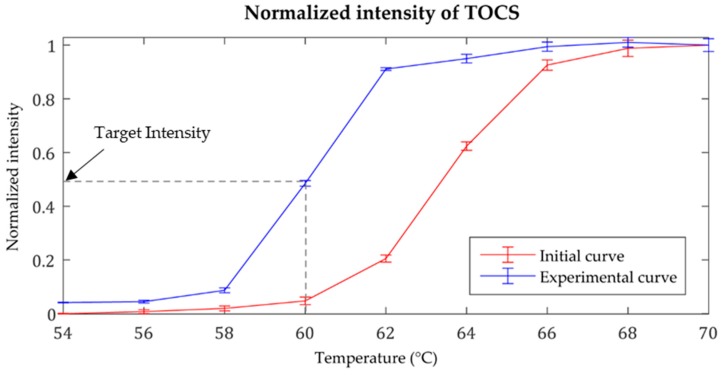
Normalized temperature-intensity curve of TOCS.

TOCS was used as the temperature sensor in our study. TOCS is a microcapsule array composed of dye, color developer, solvent, and outer capsule. When the temperature changes over the threshold value, the electron transfers between the dye (electron donor) and the color developer (electron accepter), leading to the structure changes of the dye and thus the reversible color change occur in the compound (Figure 4) [39]. We took the calibrated intensity of TOCS using the CCD camera located in the module. The normalized TOCS intensity versus temperature curve was constructed to deal with its hysteresis properties, which results in a phase mismatch at the same temperature between the cooling and heating stage. As shown in Figure 5, we determined that 50% of the maximum intensity of the experimental curve occurred at 60 °C, which matched the LAMP reaction temperature.

## 3. Results

### 3.1. Temperature Control for the LAMP Reaction

**Figure 6:**
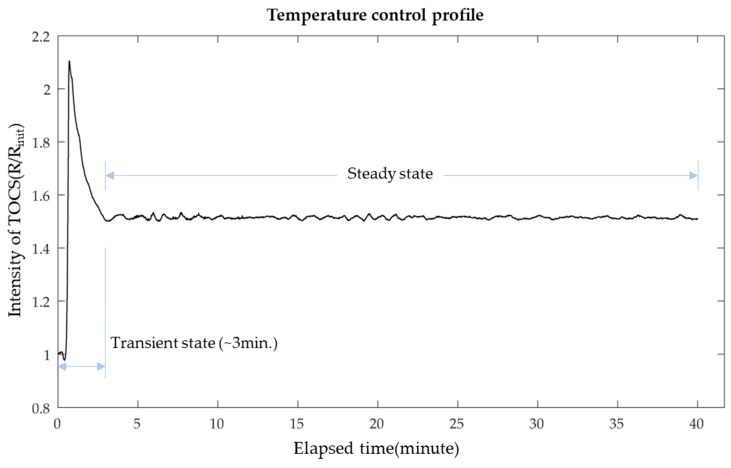
Experimental results obtained for the temperature control profile.

We measured the temperature of the LAMP reagent using TOCS for 40 min. To determine the target intensity, a 1:1 PWM duty ratio (On: 0.5 s, Off: 0.5 s) of laser emission was carried out to heat the amplification chamber until the color no longer changed and obtained its maximum value. As the maximum intensity indicated 70 °C, it does not affect the activity of polymerase. Subsequently, the program automatically calculated the target intensity, which matched 50% of the maximum value. Proportional-derivative (PD) control of laser emission duty ratio was started for the remaining 40 min following the experimental curve in order to maintain a stable heating temperature. As shown in Figure 6, PD control was successfully carried out in the suggested module, which has a mean square error of 0.1677 in the steady state (after the transient state). Considering the error in the production of each LOD amplification chamber, we scaled the TOCS intensity (R) by its initial intensity value (R_init_).

### 3.2. Analysis of the LAMP Product by CNN (Convolutional Neural Network)

**Figure 7:**
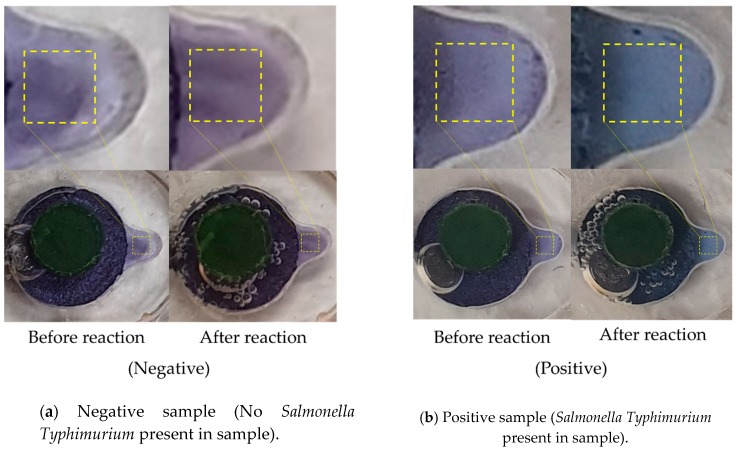
Colorimetric analysis of LAMP product using hydroxy naphthol blue (HNB), where the dotted boxes (30 × 30 pixels) indicate the region of interest (ROI) on the colorimetric detection chamber.

**Figure 8:**
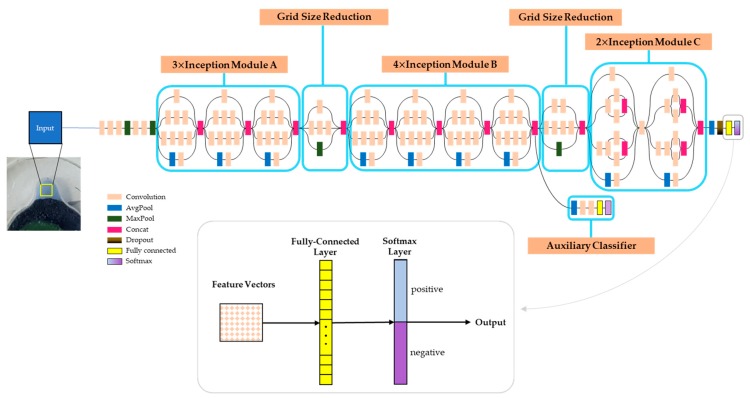
Inception-V3+ architecture [44] for colorimetric detection. It consists of 42 layers with one auxiliary classifier. The last two layers at the end were modified for transfer learning to classify LAMP product as two classes.

After amplification, the amplified DNA products were visualized using HNB dye. The amplified product changes its color in accordance with the Mg^2+^ concentration, easily distinguishable even with the naked eye when using the CCD camera located in the experimental module (Figure 7). Since its color changes from indigo to sky-blue, a simple colorimetric analysis was carried out at the end of the program by convolutional neural network [40,41]. To do this, firstly we took the colorimetric detection chamber as the region of interest (ROI). The products of LAMP assay were judged with Inception-V3 network [42,43] which is one of the most widely used CNN as image recognition model, to observe the color intensity in the ROI with high accuracy. We trained the neural network with NVIDIA GeForce GTX 1050 GPU by 100 training ROI images (Figure 8). When tested with 30 test ROI images collected from the detection chamber, the CNN achieved a classification performance of 97%.

### 3.3. LAMP Validation

**Figure 9:**
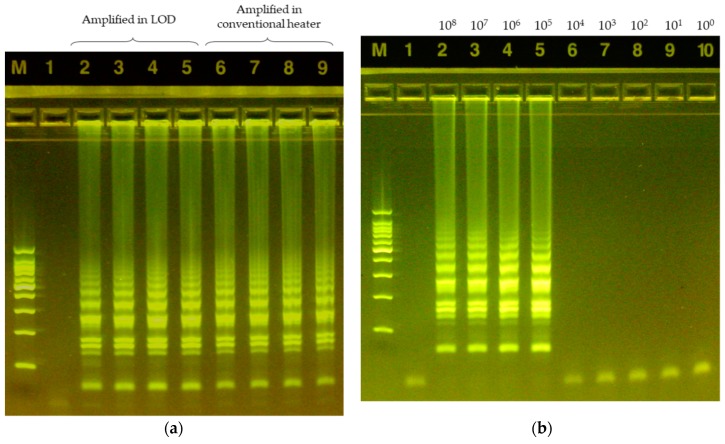
Electrophoresis result of experimental data. (**a**) M: DNA marker (24073, SizerTM-100bp DNA Marker, INtRON biotechnology, Seongnam Korea), 1st lane: Negative control (No *Salmonella* Typhimurium present in sample), 2–5th lane: LAMP product using LOD, 6–9th lane: Positive control (LAMP product using the heating incubator); (**b**) Electrophoresis result with 10-fold dilution (M: DNA marker, 1st lane: Negative control, 2nd–10th lane: LAMP product serially 10-fold diluted with the initial concentration of 10^8^ CFU/mL).

A total of four samples of the Salmonella DNA template were amplified with the target primers. Each of the samples were divided to amplify using a thermocycler and LOD. The results were visualized using HNB and validated by gel electrophoresis analysis. Figure 9a shows that the temperature control and amplification process were successfully carried out on the proposed LOD module when comparing with the conventional method. Figure 9b shows the electrophoresis result of LAMP product with various concentrations of samples. It states that the limit of detection is approximately 10^5^ CFU/mL, which was about 10 times lower in comparison to other existing methods that have been reported in the literature for detecting foodborne pathogens using LAMP [38,45]. This is because our detection simply depends on the change of color of HNB, allowing the colorimetric detection by CNN, and is rather advantageous for its portability, simplicity, and cost effectiveness.

## 4. Discussion

In this paper, we have suggested a non-contact sensor based LOD module for the colorimetric detection of LAMP using CNN. We fabricated the apparatus using a laser module, disc drive, and camera sensors for disc-based temperature control system. We automatically controlled the thermal conditions by measuring the TOCS intensity using a CCD camera with PWM control of the laser emission to the micro graphite film fabricated in the amplification chamber. The amplified product was analyzed by deep learning network for more consistent colorimetric detection than naked eye. The purpose of the study is achieved by utilizing means and heating devices inside of the LOD, which enables to heat and detecting in the manner of non-contact. Main future work includes improving the limit of detection and considering the multiple detections of various pathogens. Using the suggested method, we expect our work to be expanded and utilized in the field of POCT.

## Figures and Tables

**Table 1:** Disc driving sequence of the loop-mediated isothermal amplification (LAMP) reaction.

Step	Operation	Spinning Speed (rpm)	Duration (s)
1	Sample injection	-	-
2	Spin out to move samples in the channel	3000	30
3	Irradiate laser to LB valve	-	10
4	Spin out the residual to waste chamber	3000	10
5	Irradiate laser to melting EVA valve	-	60
6	Spin out to push the melted EVA valve in radial way	10,000	60
7	Irradiate laser to amplification chamber in adjusted duty ratio	-	2400
